# Di­chlorido­(4,4′-di-*tert*-butyl-2,2′-bi­pyridine-κ^2^ *N*,*N*′)palladium(II) dimethyl sulfoxide monosolvate monohydrate

**DOI:** 10.1107/S1600536814009453

**Published:** 2014-05-10

**Authors:** Ricardo A. Gutiérrez-Márquez, Carmela Crisóstomo-Lucas, Reyna Reyes-Martínez, Simón Hernández-Ortega, David Morales-Morales

**Affiliations:** a Instituto de Química, Universidad Nacional Autónoma de México, Circuito exterior, Ciudad Universitaria, México, DF, 04510, Mexico

## Abstract

The title compound, [PdCl_2_(C_18_H_24_N_2_)]·(CH_3_)_2_SO·H_2_O, the Pd^II^ ion is in a distorted square-planar geometry. The Pd—N bond distances are 2.022 (2) and 2.027 (2) Å, the Pd—Cl bond distances are 2.2880 (7) and 2.2833 (7) Å, and the ligand bite angle is 80.07 (9)°. The dimethyl sulfoxide and water mol­ecules form linear chains along [100] by O—H⋯O and O—H⋯S hydrogen bonds, generating eight- and 12-membered rings. C—H⋯Cl inter­actions link the chains, forming a three-dimensional arrangement. In addition, the 4,4-di-*tert*-butyl-2,2′-bi­pyridine ligand exhibits π–π stacking inter­actions [centroid–centroid distances = 3.8741 (15) and 3.8353 (15) Å]. The DMSO solvent is disordered and was refined with an occupancy ratio of 0.866 (3):0.134 (3).

## Related literature   

For compounds with N—N ligands, see: Corona-Rodríguez *et al.* (2007 ▶); Basauri-Molina *et al.* (2010 ▶). For the crystal structure of non-solvated compound, see: Qin *et al.* (2002 ▶); MacLean *et al.* (2002 ▶). For metallomacrocycles, see: Qin *et al.* (2002 ▶); Tzeng *et al.* (2001 ▶). For similar compounds and their crystal structures, see: Jones *et al.* (2007 ▶).
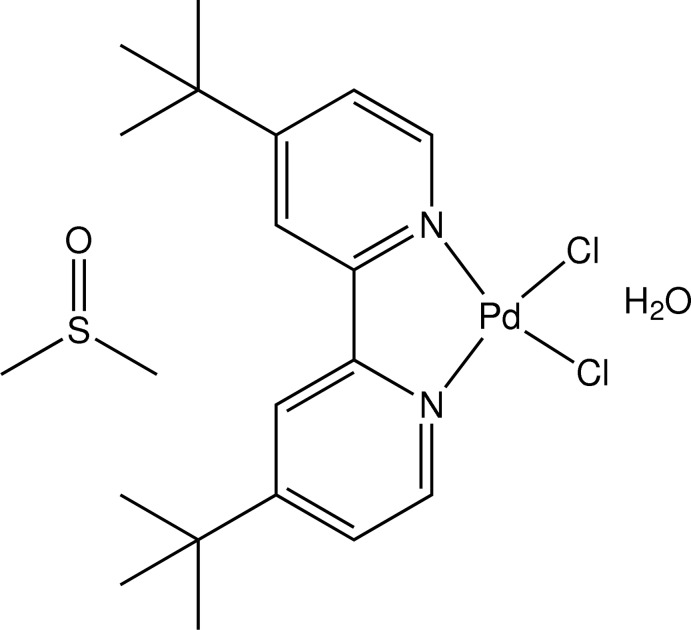


## Experimental   

### 

#### Crystal data  

[PdCl_2_(C_18_H_24_N_2_)]·C_2_H_6_OS·H_2_O
*M* *_r_* = 541.83
Monoclinic, 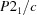

*a* = 7.4869 (3) Å
*b* = 19.5052 (8) Å
*c* = 16.8538 (7) Å
β = 102.907 (1)°
*V* = 2399.03 (17) Å^3^
*Z* = 4
Mo *K*α radiation
μ = 1.10 mm^−1^
*T* = 298 K
0.42 × 0.19 × 0.09 mm

#### Data collection  

Bruker APEXII CCD area-detector diffractometer
Absorption correction: analytical (*SADABS*; Sheldrick, 2008 ▶) *T*_min_ = 0.780, *T*_max_ = 0.932
13351 measured reflections
4343 independent reflections
3766 reflections with *I* > 2σ(*I*)
*R*_int_ = 0.030

#### Refinement  

*R*[*F* ^2^ > 2σ(*F* ^2^)] = 0.028
*wR*(*F* ^2^) = 0.076
*S* = 1.01
4343 reflections
302 parameters
118 restraints
H atoms treated by a mixture of independent and constrained refinement
Δρ_max_ = 0.39 e Å^−3^
Δρ_min_ = −0.55 e Å^−3^

### 

Data collection: *APEX2* (Bruker, 2012 ▶); cell refinement: *SAINT* (Bruker, 2012 ▶); data reduction: *SAINT*; program(s) used to solve structure: *SHELXTL* (Sheldrick, 2008 ▶); program(s) used to refine structure: *SHELXL2013* (Sheldrick, 2008 ▶); molecular graphics: *ORTEP-3 for Windows* (Farrugia, 2012 ▶) and *DIAMOND* (Brandenburg, 2006 ▶); software used to prepare material for publication: *SHELXTL* and *PLATON* (Spek, 2009 ▶).

## Supplementary Material

Crystal structure: contains datablock(s) I. DOI: 10.1107/S1600536814009453/pj2010sup1.cif

Structure factors: contains datablock(s) I. DOI: 10.1107/S1600536814009453/pj2010Isup2.hkl

Additional supporting information:  crystallographic information; 3D view; checkCIF report

## Figures and Tables

**Table 1:** Hydrogen-bond geometry (Å, °)

*D*—H⋯*A*	*D*—H	H⋯*A*	*D*⋯*A*	*D*—H⋯*A*
O2—H2*A*⋯O1^i^	0.87 (1)	2.11 (2)	2.954 (7)	165 (7)
O2—H2*A*⋯S1*A* ^i^	0.87 (1)	2.71 (2)	3.565 (14)	167 (7)
O2—H2*A*⋯O1*A* ^i^	0.87 (1)	1.68 (4)	2.51 (3)	157 (8)
O2—H2*B*⋯O1^ii^	0.87 (1)	2.20 (2)	3.054 (9)	171 (7)
O2—H2*B*⋯S1*A* ^ii^	0.87 (1)	1.84 (4)	2.604 (12)	146 (7)
O2—H2*B*⋯O1*A* ^ii^	0.87 (1)	2.30 (4)	3.17 (4)	173 (7)
C14—H14*B*⋯Cl2^iii^	0.96	2.96	3.884 (1)	163

Symmetry codes: (i) 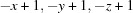; (ii) 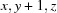; (iii) 
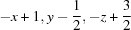
.

**Fig. 1.**
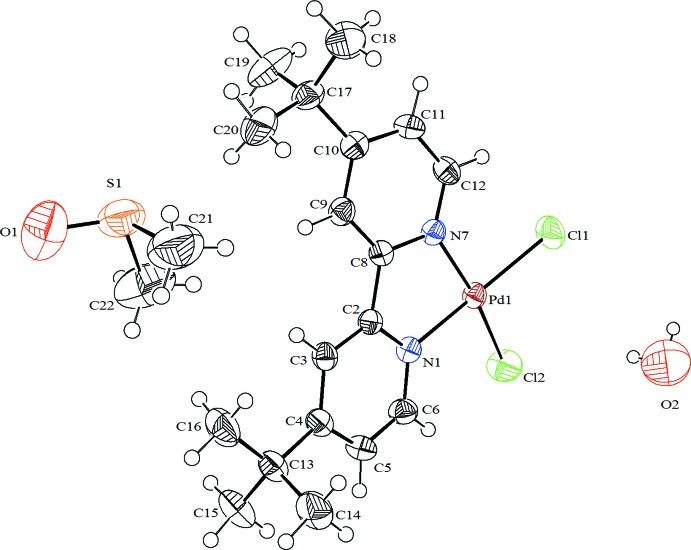
The asymmetric unit of the title compound. All non-hydrogen atoms are shown as ellipsoids with probability level of 50%.

**Fig. 2.**
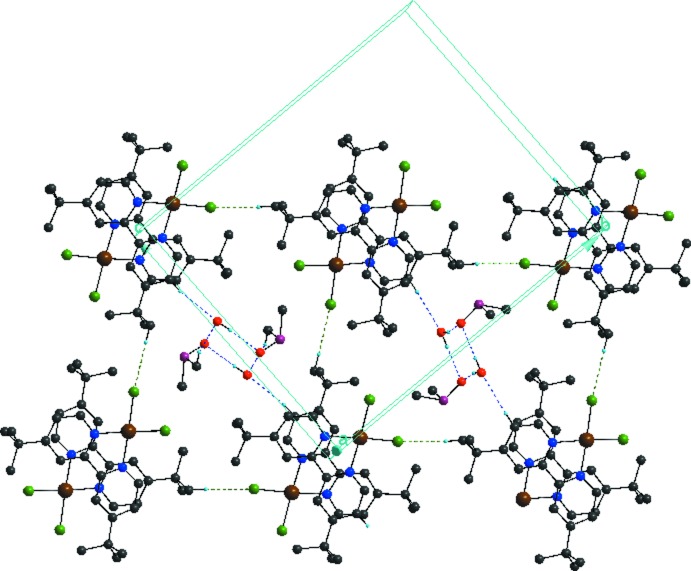
Packing of the molecular entities in the structure of the title compound. Hydrogen bonds interaction as shown by dashed lines.

## supplementary crystallographic information

### 1. Introduction

The N—N chelate ligands have been studied with a variety of transition metals as building blocks in supra­molecular chemistry. 2,2'-Bi­pyridine and its derivatives have been employed as auxiliary ligands in transition metal complexes usually serving as blocking ligands. Thus, given our continuous inter­est in the synthesis of metal complexes with potential catalytic activities in cross coupling reactions and the use of N—N quelate ligands (Corona-Rodríguez *et al.*, 2007; Basauri-Molina *et al.*, 2010), we report here the crystal structure of the compound [PdCl_2_(*^t^*Bubpy)]·(CH_3_)_2_SO)·H_2_O (*^t^*Bubpy = 4,4'-di-tert-butyl-2,2'-bi­pyridine) as a solvated compound. The crystal structure of the non-solvated complex has been reported previously (Qin *et al.*, 2002; MacLean *et al.*, 2002), and this compound has served as precursor in the formation of metallomacrocycles (Qin *et al.*, 2002; Tzeng *et al.*, 2001) and due to its structure may present π inter­actions like π-π stacking and C—H···π inter­actions.

### 2.1. Synthesis and crystallization

To a solution of [Pd(MeCN)_2_Cl_2_] (0.13 g, 0.501 mmol) in ethanol (10 ml), 4,4'-di-tert-butyl-2,2'-bi­pyridine (0.1 g, 0.651 mmol) was added under stirring. The resulting orange solution was allowed to react for 4 h under stirring at room temperature. After this time the solution was filtered and the solvent taken off under vacuum to produce a yellow solid. Crystals suitable for X-ray diffraction experiments were obtained from di­methyl­sulfoxide as solvent at room temperature.

^1^H-NMR (300 MHz, DMSO–D_6_): d 1.42 (s, 18H, ^t^Bu), 7.82 (d, 2H, CH), 8.60 (s, 2H, CH), 9.02 (d, 2H, CH). ^13^C-NMR (75.6 MHz, DMSO-D_6_): d 30.3 (s, CH_3_), 39.9 (s, C(CH_3_)_3_), 121.7 (s, CH), 124.4 (s, CH), 149.9 (s, CH), 156.4 (s, C), 165.7 (s, C).

### 2.2. Refinement

Crystal data, data collection and structure refinement details are summarized in Table 1.

H atoms were included in calculated positions (C—H = 0.93 Å for aromatic H, C—H = 0.96 Å for methyl H), and refined using a riding model with *U* iso (H) = 1.2*U*eq of the carrier atom. H atoms on O were located on the Fourier map and refined isotropically.

The DMSO solvent is disordered and was refined in two major positions using a free variable of Site Occupational Factor (SOF), the ratio of disordered atoms was 87/13 of SOF.

In the refinement six reflections, (0 0 1), (0 5 1), (1 2 2), (1 2 0), (-1 2 4) and (-2 0 2), were considered as disagreeable and were omitted.

### 3. Results and discussion

The title compound is formed by a molecule of the complex [PdCl_2_(*^t^*Bubpy)], one molecule of di­methyl­sulfoxide (disordered) and one molecule of water, the structure is presented in Figure 1.

The coordination around the Pd(II) ion adopts a distorted square planar geometry, surrounded by two chloride atoms and one 4,4'-di-tert-butyl-2,2'-bi­pyridine ligand coordinated in a chelate fashion with a bite angle of 80.07 (8)°. The Pd—Cl bond distances are 2.2880 (9) and 2.2832 (9) Å which are similar to those found in the non-solvated structure (Qin *et al.*, 2002; MacLean *et al.*, 2002). The Pd—N bond distances are 2.022 (2) and 2.027 (2) Å which are close in value to those found for the compound [PdCl_2_(*^t^*Bubpy)] and slightly smaller than those observed in the di­iodo complex [PdI_2_(*^t^*Bubpy)] (2.047 (4), 2.062 (4) Å) (Jones *et al.*, 2007).

Both the di­methyl­sulfoxide and water molecules inter­act *via* hydrogen bonds (O—H···O, C—H···O) forming 8- and 12-member rings that generate a linear chain in the [100] direction (Table 1). The 4,4'-di-tert-butyl-2,2'-bi­pyridine ligand presents π-π stacking inter­actions which extend along the *a* axis [100] generated by the symmetry operations 1-*x*, 1-*y*, 1-*z* and -*x*, 1-*y*, 1-*z*. The two centroid-centroid distances between the ligand rings are 3.8741 (15) and 3.8353 (15) Å respectively. The crystal arrangement is complemented by C—H···Cl inter­actions which link the linear arrangement of π-π stacking in layers parallel to (100).

### Crystal data

**Table:**

[PdCl_2_(C_18_H_24_N_2_)]·C_2_H_6_OS·H_2_O	*F*(000) = 1112
*M**_r_* = 541.83	*D*_x_ = 1.500 Mg m^−^^3^
Monoclinic, *P*2_1_/*c*	Mo *K*α radiation, λ = 0.71073 Å
*a* = 7.4869 (3) Å	Cell parameters from 9545 reflections
*b* = 19.5052 (8) Å	θ = 2.4–25.3°
*c* = 16.8538 (7) Å	µ = 1.10 mm^−^^1^
β = 102.907 (1)°	*T* = 298 K
*V* = 2399.03 (17) Å^3^	Prism, yellow
*Z* = 4	0.42 × 0.19 × 0.09 mm

### Data collection

**Table:**

Bruker APEXII CCD area-detector diffractometer	3766 reflections with *I* > 2σ(*I*)
Detector resolution: 0.83 pixels mm^-1^	*R*_int_ = 0.030
ω scans	θ_max_ = 25.3°, θ_min_ = 2.1°
Absorption correction: analytical (*SADABS*; Sheldrick, 2008)	*h* = −9→8
*T*_min_ = 0.780, *T*_max_ = 0.932	*k* = −22→23
13351 measured reflections	*l* = −20→17
4343 independent reflections	

### Refinement

**Table:**

Refinement on *F*^2^	118 restraints
Least-squares matrix: full	Hydrogen site location: mixed
*R*[*F*^2^ > 2σ(*F*^2^)] = 0.028	H atoms treated by a mixture of independent and constrained refinement
*wR*(*F*^2^) = 0.076	*w* = 1/[σ^2^(*F*_o_^2^) + (0.042*P*)^2^ + 0.5*P*] where *P* = (*F*_o_^2^ + 2*F*_c_^2^)/3
*S* = 1.01	(Δ/σ)_max_ = 0.002
4343 reflections	Δρ_max_ = 0.39 e Å^−^^3^
302 parameters	Δρ_min_ = −0.55 e Å^−^^3^

### Special details

**Table:**

Geometry. All e.s.d.'s (except the e.s.d. in the dihedral angle between two l.s. planes) are estimated using the full covariance matrix. The cell e.s.d.'s are taken into account individually in the estimation of e.s.d.'s in distances, angles and torsion angles; correlations between e.s.d.'s in cell parameters are only used when they are defined by crystal symmetry. An approximate (isotropic) treatment of cell e.s.d.'s is used for estimating e.s.d.'s involving l.s. planes.

### Fractional atomic coordinates and isotropic or equivalent isotropic displacement parameters (Å^2^)

**Table:**

	*x*	*y*	*z*	*U*_iso_*/*U*_eq_	Occ. (<1)
Pd1	0.23053 (3)	0.61408 (2)	0.50569 (2)	0.02992 (9)	
Cl1	0.06474 (11)	0.69947 (4)	0.42999 (5)	0.0527 (2)	
Cl2	0.38522 (12)	0.69340 (4)	0.59479 (5)	0.0573 (2)	
N1	0.3456 (3)	0.53217 (10)	0.57115 (12)	0.0297 (5)	
C2	0.3043 (3)	0.47048 (12)	0.53503 (14)	0.0282 (5)	
C3	0.3586 (3)	0.40999 (13)	0.57591 (15)	0.0325 (6)	
H3	0.3309	0.3684	0.5490	0.039*	
C4	0.4548 (3)	0.41033 (14)	0.65727 (15)	0.0342 (6)	
C5	0.4957 (4)	0.47497 (14)	0.69216 (16)	0.0378 (6)	
H5	0.5611	0.4783	0.7459	0.045*	
C6	0.4416 (4)	0.53308 (14)	0.64896 (15)	0.0373 (6)	
H6	0.4721	0.5752	0.6741	0.045*	
N7	0.1298 (3)	0.53767 (10)	0.42684 (12)	0.0296 (5)	
C8	0.1932 (3)	0.47392 (12)	0.45083 (14)	0.0274 (5)	
C9	0.1545 (3)	0.41861 (13)	0.39897 (15)	0.0337 (6)	
H9	0.1980	0.3755	0.4173	0.040*	
C10	0.0514 (3)	0.42587 (13)	0.31945 (15)	0.0333 (6)	
C11	−0.0115 (4)	0.49177 (14)	0.29707 (15)	0.0375 (6)	
H11	−0.0813	0.4996	0.2449	0.045*	
C12	0.0281 (3)	0.54553 (13)	0.35099 (15)	0.0358 (6)	
H12	−0.0171	0.5888	0.3343	0.043*	
C13	0.5026 (4)	0.34551 (14)	0.70770 (17)	0.0407 (6)	
C14	0.3815 (5)	0.34392 (17)	0.7700 (2)	0.0599 (9)	
H14A	0.4021	0.3846	0.8028	0.072*	
H14B	0.4114	0.3043	0.8043	0.072*	
H14C	0.2550	0.3418	0.7420	0.072*	
C15	0.7021 (4)	0.34643 (18)	0.7528 (2)	0.0623 (9)	
H15A	0.7790	0.3444	0.7142	0.075*	
H15B	0.7266	0.3076	0.7885	0.075*	
H15C	0.7270	0.3879	0.7840	0.075*	
C16	0.4686 (5)	0.28042 (15)	0.6560 (2)	0.0601 (9)	
H16A	0.3408	0.2772	0.6303	0.072*	
H16B	0.5042	0.2411	0.6901	0.072*	
H16C	0.5394	0.2821	0.6150	0.072*	
C17	0.0188 (4)	0.36691 (14)	0.25874 (17)	0.0419 (7)	
C18	−0.1755 (5)	0.36822 (19)	0.2081 (2)	0.0730 (11)	
H18A	−0.2600	0.3662	0.2433	0.088*	
H18B	−0.1948	0.3295	0.1720	0.088*	
H18C	−0.1951	0.4098	0.1768	0.088*	
C19	0.1540 (6)	0.3743 (2)	0.2040 (2)	0.0750 (12)	
H19A	0.1374	0.4181	0.1774	0.090*	
H19B	0.1331	0.3386	0.1638	0.090*	
H19C	0.2768	0.3709	0.2362	0.090*	
C20	0.0506 (5)	0.29733 (16)	0.3010 (2)	0.0643 (10)	
H20A	0.1775	0.2929	0.3277	0.077*	
H20B	0.0180	0.2615	0.2613	0.077*	
H20C	−0.0237	0.2938	0.3404	0.077*	
S1	0.13101 (19)	0.11340 (6)	0.41808 (7)	0.0740 (4)	0.866 (3)
O1	0.2143 (7)	0.0432 (3)	0.4267 (4)	0.1113 (14)	0.866 (3)
C21	−0.0510 (7)	0.1118 (3)	0.4701 (4)	0.0994 (19)	0.866 (3)
H21A	−0.1488	0.0837	0.4405	0.119*	0.866 (3)
H21B	−0.0951	0.1576	0.4742	0.119*	0.866 (3)
H21C	−0.0074	0.0933	0.5237	0.119*	0.866 (3)
C22	0.2808 (10)	0.1680 (3)	0.4875 (4)	0.112 (2)	0.866 (3)
H22A	0.3915	0.1751	0.4688	0.135*	0.866 (3)
H22B	0.3099	0.1469	0.5404	0.135*	0.866 (3)
H22C	0.2219	0.2112	0.4908	0.135*	0.866 (3)
S1A	0.2169 (17)	0.0735 (7)	0.4816 (7)	0.114 (3)	0.134 (3)
O1A	0.258 (4)	0.0344 (17)	0.411 (2)	0.113 (5)	0.134 (3)
C21A	−0.028 (2)	0.0783 (19)	0.463 (2)	0.094 (5)	0.134 (3)
H21D	−0.0771	0.0334	0.4678	0.112*	0.134 (3)
H21E	−0.0754	0.0953	0.4086	0.112*	0.134 (3)
H21F	−0.0628	0.1086	0.5015	0.112*	0.134 (3)
C22A	0.252 (5)	0.1620 (9)	0.462 (3)	0.107 (6)	0.134 (3)
H22D	0.3807	0.1706	0.4682	0.128*	0.134 (3)
H22E	0.2053	0.1899	0.4996	0.128*	0.134 (3)
H22F	0.1890	0.1730	0.4073	0.128*	0.134 (3)
O2	0.4246 (8)	1.0021 (3)	0.5968 (3)	0.1554 (16)	
H2A	0.522 (6)	0.990 (4)	0.580 (4)	0.187*	
H2B	0.377 (10)	1.015 (4)	0.5472 (18)	0.187*	

### Atomic displacement parameters (Å^2^)

**Table:**

	*U*^11^	*U*^22^	*U*^33^	*U*^12^	*U*^13^	*U*^23^
Pd1	0.03419 (14)	0.02250 (12)	0.03379 (13)	−0.00049 (8)	0.00909 (9)	−0.00093 (7)
Cl1	0.0637 (5)	0.0305 (4)	0.0592 (5)	0.0118 (3)	0.0034 (4)	0.0048 (3)
Cl2	0.0757 (6)	0.0323 (4)	0.0567 (5)	−0.0099 (4)	−0.0007 (4)	−0.0110 (3)
N1	0.0307 (11)	0.0271 (11)	0.0323 (11)	−0.0022 (9)	0.0088 (9)	−0.0002 (9)
C2	0.0255 (12)	0.0305 (13)	0.0293 (12)	−0.0005 (10)	0.0076 (10)	0.0003 (10)
C3	0.0354 (14)	0.0260 (13)	0.0362 (14)	0.0010 (11)	0.0085 (12)	0.0021 (11)
C4	0.0300 (14)	0.0382 (14)	0.0352 (14)	0.0019 (11)	0.0088 (11)	0.0066 (12)
C5	0.0376 (15)	0.0417 (16)	0.0307 (13)	−0.0020 (12)	0.0005 (12)	0.0037 (12)
C6	0.0420 (16)	0.0359 (15)	0.0322 (13)	−0.0025 (12)	0.0043 (12)	−0.0019 (11)
N7	0.0322 (11)	0.0267 (11)	0.0311 (11)	−0.0001 (9)	0.0097 (9)	0.0005 (9)
C8	0.0264 (13)	0.0269 (13)	0.0299 (12)	−0.0012 (10)	0.0086 (10)	0.0026 (10)
C9	0.0381 (15)	0.0271 (13)	0.0363 (14)	−0.0003 (11)	0.0094 (12)	−0.0007 (11)
C10	0.0297 (14)	0.0356 (15)	0.0355 (13)	−0.0055 (11)	0.0088 (11)	−0.0029 (11)
C11	0.0370 (15)	0.0424 (16)	0.0294 (13)	−0.0016 (12)	−0.0007 (12)	0.0018 (12)
C12	0.0361 (15)	0.0314 (14)	0.0383 (14)	0.0022 (11)	0.0051 (12)	0.0067 (11)
C13	0.0383 (15)	0.0392 (16)	0.0434 (16)	0.0051 (13)	0.0068 (13)	0.0109 (13)
C14	0.066 (2)	0.057 (2)	0.062 (2)	0.0104 (17)	0.0274 (18)	0.0289 (17)
C15	0.0480 (19)	0.062 (2)	0.071 (2)	0.0108 (17)	−0.0007 (17)	0.0235 (18)
C16	0.068 (2)	0.0397 (17)	0.068 (2)	0.0097 (16)	0.0046 (18)	0.0151 (16)
C17	0.0435 (17)	0.0425 (16)	0.0383 (15)	−0.0064 (13)	0.0059 (13)	−0.0109 (13)
C18	0.060 (2)	0.064 (2)	0.080 (3)	−0.0053 (18)	−0.015 (2)	−0.031 (2)
C19	0.089 (3)	0.083 (3)	0.062 (2)	−0.026 (2)	0.037 (2)	−0.036 (2)
C20	0.083 (3)	0.0400 (18)	0.066 (2)	−0.0046 (17)	0.010 (2)	−0.0193 (16)
S1	0.0947 (10)	0.0791 (9)	0.0486 (6)	−0.0027 (6)	0.0171 (6)	−0.0019 (5)
O1	0.129 (4)	0.086 (2)	0.125 (4)	0.013 (2)	0.040 (3)	−0.020 (3)
C21	0.108 (4)	0.129 (6)	0.065 (3)	0.002 (3)	0.026 (3)	−0.006 (4)
C22	0.144 (5)	0.115 (4)	0.078 (4)	−0.035 (4)	0.024 (4)	−0.025 (3)
S1A	0.138 (5)	0.114 (6)	0.085 (5)	−0.003 (5)	0.010 (5)	0.002 (5)
O1A	0.123 (10)	0.108 (9)	0.104 (10)	0.005 (10)	0.015 (9)	−0.005 (8)
C21A	0.135 (6)	0.085 (11)	0.058 (10)	0.006 (8)	0.016 (9)	0.010 (11)
C22A	0.140 (11)	0.113 (7)	0.066 (12)	−0.012 (9)	0.020 (11)	−0.009 (9)
O2	0.173 (5)	0.174 (4)	0.126 (3)	0.025 (4)	0.047 (3)	0.023 (3)

### Geometric parameters (Å, º)

**Table:**

Pd1—N1	2.022 (2)	C16—H16B	0.9600
Pd1—N7	2.027 (2)	C16—H16C	0.9600
Pd1—Cl2	2.2833 (7)	C17—C18	1.513 (4)
Pd1—Cl1	2.2880 (7)	C17—C19	1.522 (4)
N1—C6	1.347 (3)	C17—C20	1.526 (4)
N1—C2	1.353 (3)	C18—H18A	0.9600
C2—C3	1.381 (3)	C18—H18B	0.9600
C2—C8	1.477 (3)	C18—H18C	0.9600
C3—C4	1.399 (3)	C19—H19A	0.9600
C3—H3	0.9300	C19—H19B	0.9600
C4—C5	1.396 (4)	C19—H19C	0.9600
C4—C13	1.521 (4)	C20—H20A	0.9600
C5—C6	1.359 (4)	C20—H20B	0.9600
C5—H5	0.9300	C20—H20C	0.9600
C6—H6	0.9300	S1—O1	1.498 (5)
N7—C12	1.342 (3)	S1—C21	1.777 (5)
N7—C8	1.360 (3)	S1—C22	1.782 (5)
C8—C9	1.378 (3)	C21—H21A	0.9600
C9—C10	1.396 (3)	C21—H21B	0.9600
C9—H9	0.9300	C21—H21C	0.9600
C10—C11	1.392 (4)	C22—H22A	0.9600
C10—C17	1.522 (4)	C22—H22B	0.9600
C11—C12	1.376 (4)	C22—H22C	0.9600
C11—H11	0.9300	S1A—O1A	1.504 (12)
C12—H12	0.9300	S1A—C22A	1.789 (9)
C13—C15	1.517 (4)	S1A—C21A	1.792 (9)
C13—C16	1.529 (4)	C21A—H21D	0.9600
C13—C14	1.534 (4)	C21A—H21E	0.9600
C14—H14A	0.9600	C21A—H21F	0.9600
C14—H14B	0.9600	C22A—H22D	0.9600
C14—H14C	0.9600	C22A—H22E	0.9600
C15—H15A	0.9600	C22A—H22F	0.9600
C15—H15B	0.9600	O2—H2A	0.869 (10)
C15—H15C	0.9600	O2—H2B	0.867 (10)
C16—H16A	0.9600		
			
N1—Pd1—N7	80.07 (9)	C13—C16—H16B	109.5
N1—Pd1—Cl2	94.87 (6)	H16A—C16—H16B	109.5
N7—Pd1—Cl2	171.60 (6)	C13—C16—H16C	109.5
N1—Pd1—Cl1	172.53 (6)	H16A—C16—H16C	109.5
N7—Pd1—Cl1	95.37 (6)	H16B—C16—H16C	109.5
Cl2—Pd1—Cl1	90.33 (3)	C18—C17—C19	110.0 (3)
C6—N1—C2	117.9 (2)	C18—C17—C10	110.9 (2)
C6—N1—Pd1	126.19 (17)	C19—C17—C10	107.9 (2)
C2—N1—Pd1	115.51 (16)	C18—C17—C20	108.0 (3)
N1—C2—C3	121.5 (2)	C19—C17—C20	108.2 (3)
N1—C2—C8	114.5 (2)	C10—C17—C20	111.9 (2)
C3—C2—C8	123.9 (2)	C17—C18—H18A	109.5
C2—C3—C4	121.0 (2)	C17—C18—H18B	109.5
C2—C3—H3	119.5	H18A—C18—H18B	109.5
C4—C3—H3	119.5	C17—C18—H18C	109.5
C5—C4—C3	115.7 (2)	H18A—C18—H18C	109.5
C5—C4—C13	120.9 (2)	H18B—C18—H18C	109.5
C3—C4—C13	123.3 (2)	C17—C19—H19A	109.5
C6—C5—C4	121.1 (2)	C17—C19—H19B	109.5
C6—C5—H5	119.5	H19A—C19—H19B	109.5
C4—C5—H5	119.5	C17—C19—H19C	109.5
N1—C6—C5	122.7 (2)	H19A—C19—H19C	109.5
N1—C6—H6	118.6	H19B—C19—H19C	109.5
C5—C6—H6	118.6	C17—C20—H20A	109.5
C12—N7—C8	118.3 (2)	C17—C20—H20B	109.5
C12—N7—Pd1	126.10 (17)	H20A—C20—H20B	109.5
C8—N7—Pd1	115.04 (16)	C17—C20—H20C	109.5
N7—C8—C9	121.1 (2)	H20A—C20—H20C	109.5
N7—C8—C2	114.3 (2)	H20B—C20—H20C	109.5
C9—C8—C2	124.6 (2)	O1—S1—C21	106.5 (3)
C8—C9—C10	121.5 (2)	O1—S1—C22	107.1 (3)
C8—C9—H9	119.3	C21—S1—C22	97.2 (3)
C10—C9—H9	119.3	S1—C21—H21A	109.5
C11—C10—C9	115.8 (2)	S1—C21—H21B	109.5
C11—C10—C17	121.4 (2)	H21A—C21—H21B	109.5
C9—C10—C17	122.7 (2)	S1—C21—H21C	109.5
C12—C11—C10	121.0 (2)	H21A—C21—H21C	109.5
C12—C11—H11	119.5	H21B—C21—H21C	109.5
C10—C11—H11	119.5	S1—C22—H22A	109.5
N7—C12—C11	122.3 (2)	S1—C22—H22B	109.5
N7—C12—H12	118.8	H22A—C22—H22B	109.5
C11—C12—H12	118.8	S1—C22—H22C	109.5
C15—C13—C4	110.6 (2)	H22A—C22—H22C	109.5
C15—C13—C16	108.4 (3)	H22B—C22—H22C	109.5
C4—C13—C16	112.5 (2)	O1A—S1A—C22A	106.1 (8)
C15—C13—C14	108.9 (3)	O1A—S1A—C21A	105.7 (8)
C4—C13—C14	107.3 (2)	C22A—S1A—C21A	96.0 (7)
C16—C13—C14	109.0 (3)	S1A—C21A—H21D	109.5
C13—C14—H14A	109.5	S1A—C21A—H21E	109.5
C13—C14—H14B	109.5	H21D—C21A—H21E	109.5
H14A—C14—H14B	109.5	S1A—C21A—H21F	109.5
C13—C14—H14C	109.5	H21D—C21A—H21F	109.5
H14A—C14—H14C	109.5	H21E—C21A—H21F	109.5
H14B—C14—H14C	109.5	S1A—C22A—H22D	109.5
C13—C15—H15A	109.5	S1A—C22A—H22E	109.5
C13—C15—H15B	109.5	H22D—C22A—H22E	109.5
H15A—C15—H15B	109.5	S1A—C22A—H22F	109.5
C13—C15—H15C	109.5	H22D—C22A—H22F	109.5
H15A—C15—H15C	109.5	H22E—C22A—H22F	109.5
H15B—C15—H15C	109.5	H2A—O2—H2B	88 (6)
C13—C16—H16A	109.5		

### Hydrogen-bond geometry (Å, º)

**Table:**

*D*—H···*A*	*D*—H	H···*A*	*D*···*A*	*D*—H···*A*
O2—H2*A*···O1^i^	0.87 (1)	2.11 (2)	2.954 (7)	165 (7)
O2—H2*A*···S1*A*^i^	0.87 (1)	2.71 (2)	3.565 (14)	167 (7)
O2—H2*A*···O1*A*^i^	0.87 (1)	1.68 (4)	2.51 (3)	157 (8)
O2—H2*B*···O1^ii^	0.87 (1)	2.20 (2)	3.054 (9)	171 (7)
O2—H2*B*···S1*A*^ii^	0.87 (1)	1.84 (4)	2.604 (12)	146 (7)
O2—H2*B*···O1*A*^ii^	0.87 (1)	2.30 (4)	3.17 (4)	173 (7)
C14—H14*B*···Cl2^iii^	0.96	2.96	3.884 (1)	163

Symmetry codes: (i) −*x*+1, −*y*+1, −*z*+1; (ii) *x*, *y*+1, *z*; (iii) −*x*+1, *y*−1/2, −*z*+3/2.
